# Effective venetoclax-based treatment in relapsed/refractory multiple myeloma patients with translocation t(6;14)

**DOI:** 10.3389/pore.2023.1611375

**Published:** 2023-11-10

**Authors:** Andrea Ceglédi, Zoltán Csukly, Mónika Fekete, András Kozma, Zsuzsanna Szemlaky, Hajnalka Andrikovics, Gábor Mikala

**Affiliations:** ^1^ Department of Hematology and Stem Cell Transplantation, Central Hospital of Southern Pest, National Institute for Hematology and Infectious Diseases, Budapest, Hungary; ^2^ Department of Public Health, Semmelweis University, Budapest, Hungary; ^3^ Laboratory of Molecular Genetics, Central Hospital of Southern Pest, National Institute for Hematology and Infectious Diseases, Budapest, Hungary

**Keywords:** multiple myeloma, venetoclax, translocation t(6;14), IGH::CCND3, personalized therapy

## Abstract

**Introduction*:*** The selective Bcl-2 inhibitor venetoclax has shown promising therapeutic potential in multiple myeloma, particularly in cases associated with t(11;14) *IGH::CCND1* translocation. However, the efficacy of venetoclax in myeloma patients with the t(6;14) *IGH::CCND3* translocation remains less investigated.

**Methods:** In this study, we conducted a retrospective analysis to investigate the efficacy of venetoclax-based therapy in relapsed/refractory myeloma patients with t(6;14) translocation. The treatment courses of three patients, that included previous therapies and responses to venetoclax, were assessed. Clinical data, laboratory results, and adverse events were analyzed to evaluate treatment outcomes.

**Results:** Our findings demonstrated remarkable therapeutic responses in three consecutive patients with t(6;14) translocation-associated myeloma who received venetoclax-based therapy. Patient 1, a lenalidomide-bortezomib-daratumumab and alkylator treatment refractory patient, achieved sustained stringent complete remission (sCR) after combining carfilzomib-dexamethasone with venetoclax, which was his best response ever. Similarly, Patient 2, refractory to frontline bortezomib-thalidomide-dexamethasone therapy, attained CR following a transition to bortezomib-dexamethason-venetoclax treatment. Patient 3, who was immunomodulatory (IMID)-intolerant, showed a highly favorable response to venetoclax-dexamethasone therapy after his first relapse following autologous stem cell transplantation. No significant adverse effects were observed in any of the patients.

**Discussion:** Our study provides compelling preliminary evidence for the efficacy of venetoclax in t(6;14) translocation-associated myeloma. The outcomes observed in our patients suggest that venetoclax-based therapy holds substantial promise as an effective treatment option for this specific genetic subgroup. Furthermore, the similarities in treatment response between t(11;14) and t(6;14) translocation subgroups highlight the importance of personalized approaches targeting specific genetic abnormalities to optimize therapeutic outcomes.

## Introduction

Multiple myeloma is a complex hematological malignancy characterized by the clonal proliferation of plasma cells in the bone marrow. It is considered a quintessential disease of aging, as it is typically diagnosed at an average age of 70 years [1]. The incidence of multiple myeloma significantly increases with advancing age, making it more prevalent in older individuals [1]. Despite the challenges associated with an aging population, advancements in treatment options have significantly improved outcomes for patients with multiple myeloma. Among these therapeutic advancements, the selective Bcl-2 inhibitor venetoclax has emerged as a promising avenue, demonstrating notable efficacy in multiple myeloma patients. This efficacy has been highlighted by the landmark BELLINI study, which investigated the use of venetoclax-based therapy for multiple myeloma [2]. It revealed encouraging results, demonstrating favorable response rates and improved progression-free survival in patients receiving venetoclax in combination with standard treatment regimens [2]. This promising efficacy has positioned venetoclax as an attractive addition to the therapeutic armamentarium against multiple myeloma.

Despite the therapeutic benefits observed with venetoclax [2–18], concerns have emerged regarding its safety profile, particularly in relation to infectious complications [19–21]. Subsequent investigations have indicated a higher incidence of infectious mortality within the venetoclax treatment groups, raising important questions regarding patient selection and risk management strategies. As such, there is an urgent need to identify and carefully select patients who are most likely to derive substantial benefits from venetoclax therapy while minimizing the potential risks associated with infectious complications.

The expression of Bcl-2, a key anti-apoptotic protein and the molecular target of venetoclax, has emerged as an important predictor of therapeutic response to venetoclax in myeloma patients. Multiple myeloma is characterized by significant genomic heterogeneity [22]. Expression profiles of Bcl-2 family members can vary significantly across multiple myeloma patients with different genetic backgrounds, emphasizing the distinct genetic subtypes that exist. Notably, the t(11;14) translocation has been commonly associated with elevated Bcl-2 expression, serving as a potential genetic marker for identifying patients who may particularly benefit from venetoclax treatment [2–4]. However, routine diagnostic measurement of Bcl-2 expression levels remains a challenge, hindering its widespread use in clinical practice. Further investigation into the relationship between genetic subtypes and Bcl-2 expression patterns holds great promise for refining patient selection strategies and optimizing the efficacy of venetoclax in the management of multiple myeloma.

Recognizing the striking similarity in gene expression profiles between patients with t(11;14) and t(6;14) translocations [23–25], our recent endeavors have focused on implementing venetoclax-based therapy specifically for patients harboring the t(6; 14) translocation. This decision finds support in studies, which extensively compared the transcriptomes of multiple myeloma cells across diverse genetic backgrounds [25]. Analysis of the molecular landscape of various genetic subtypes underscores the noteworthy presence of elevated Bcl-2 expression and thus the potential effectiveness of venetoclax in t(6;14) translocation-associated multiple myeloma. By leveraging this knowledge, our objective is to broaden treatment options for patients with the t(6;14) translocation, fostering personalized approaches that specifically target genetic abnormalities to optimize therapeutic outcomes.

Within this context, our present pilot study aims to investigate the safety and efficacy of venetoclax in a distinct subset of multiple myeloma patients. By analyzing the clinical data of a meticulously selected relapsed/refractory patient cohort exhibiting the t(6;14) translocation, our study seeks to elucidate the therapeutic potential of venetoclax for this particular genetic subtype. Furthermore, our research endeavors to provide valuable insights into the identification and selection of patients who are most suited to receive venetoclax-based therapy, thus maximizing therapeutic outcomes while ensuring that their safety remains paramount.

## Methods

We conducted a retrospective analysis of the medical records of three patients diagnosed with multiple myeloma characterized by the presence of the t(6;14) founding translocation, who received treatment that included venetoclax. The publication of this report was approved by the ethics committee of Central Hospital of South Pest/National Institute of Hematology and Infectious Diseases, and written informed consent was obtained from the patients. Each individual therapy was a preapproved “off-label” therapy authorized by the Hungarian National Institute for Pharmacy and Public Health (OGYÉI/22147-2/2021).

Data were collected from the patient’s medical records, which included comprehensive information such as laboratory test results, imaging studies, surgical reports, and medication administration records. The patients’ clinical courses were meticulously tracked from their initial admission to our department up to the present day. Descriptive statistics were employed to analyze the patient’s clinical data, including laboratory test results and medication regimens. The changes in M protein and λFLC (free light chain) over time were graphically depicted to illustrate the treatment response. Assessment of therapy response additionally encompassed monitoring clinical symptoms, laboratory test results, and the occurrence of adverse events.

## Results

### Case report: patient #1

Patient Information: A 56 year-old male was diagnosed with IgG kappa myeloma in June 2013. He presented with an International Staging System (ISS) score of III and demonstrated the *IGH::CCND3* translocation and monosomy 13 based on fluorescence *in situ* hybridization (FISH) analysis.

**FIGURE 1:**
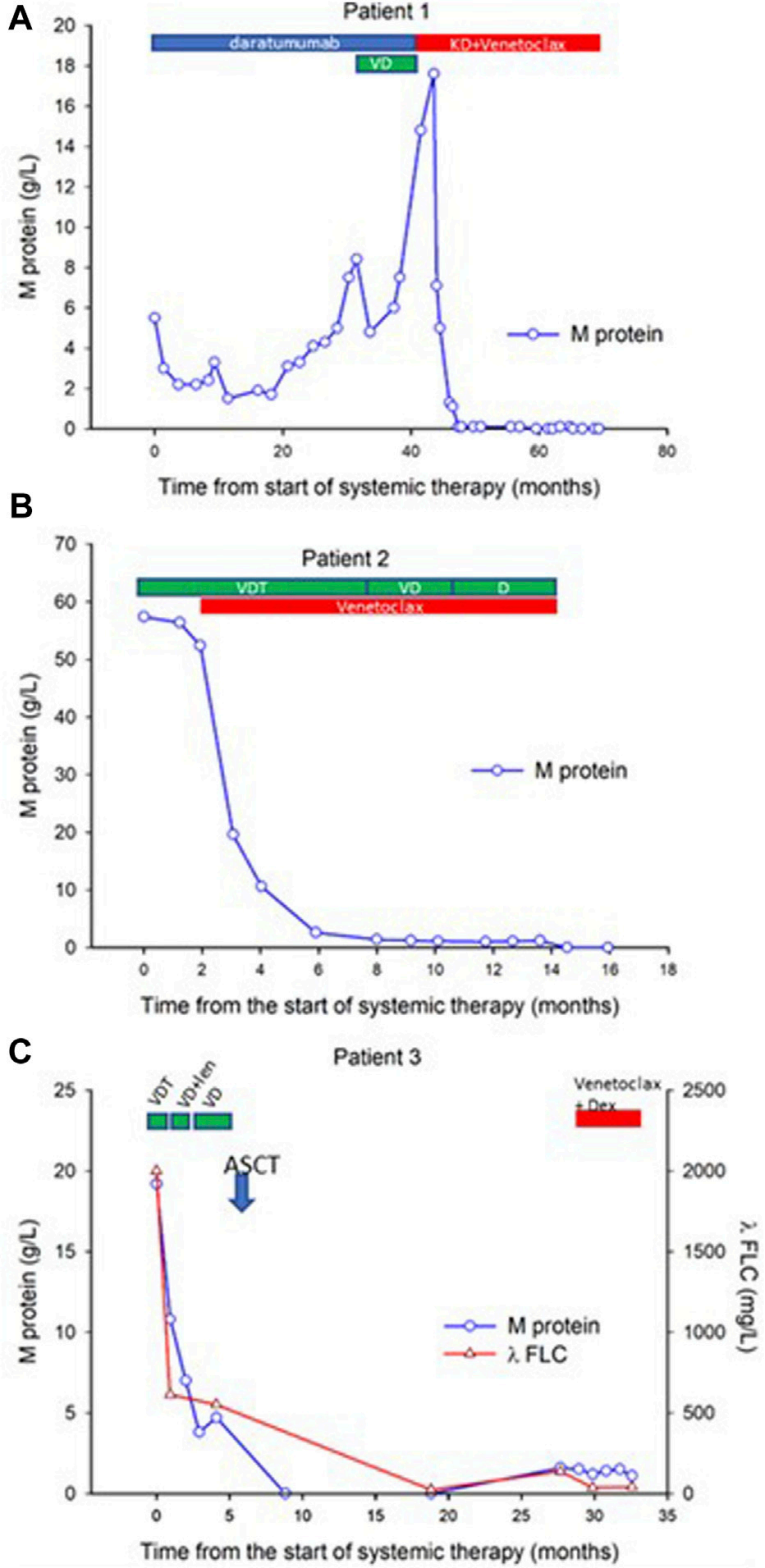
Time course of venetoclax therapy-induced changes in M protein in three myeloma patients. Sequential serum concentrations of M protein throughout the disease course are presented for three patients. Panels **(A–C)** depict the individual patients (patients 1, 2, and 3, respectively), showcasing the treatment type and duration (Patient #1 is treated for 24 months, patient #2 is treated for 12 months, patient #3 is treated for 6 months, therapies for patients #2 and #3 are currently ongoing, patient #1 is in treatment-free remission). Patient 3 results also include serial measurements of λ FLC to further demonstrate treatment response. VDT: Bortezomib, Dexamethasone, and Thalidomide; VD, Bortezomib, Dexamethasone; D, Dexamathasone; ASCT, Autologous Stem Cell Transplantation; Len, Lenalidomide; KD, Carfilzomib and Dexamethasone.

Treatment History: The patient underwent multiple treatment lines over the course of his disease. From 06/14/2013 he received 5 cycles of VDT (combination therapy with bortezomib, dexamethasone, and thalidomide), followed by high-dose cyclophosphamide (HDCy priming; 6,000 mg) mobilization and autologous stem cell transplantation (ASCT; in 05/20/2015; 400 mg Melphalan). In subsequent treatment lines, the patient received 4 cycles of Len-Dex (lenalidomide and dexamethasone). The 5th and 6th cycles of Len-Dex were unsuccessfully combined with *per os* Melphalan treatment. In 2017, he was administered daratumumab monotherapy, followed by daratumumab, bortezomib, and dexamethasone triplet treatment in 2020. Despite the various treatment approaches, the patient’s myeloma remained refractory to available therapies, as evidenced by the serial measurements of the M protein, a key marker of myeloma (Figure 1A). He also developed a paramedullary plasmacytoma in the cervical spine, further complicating his medical condition. The clinical situation became increasingly desperate, prompting a thorough evaluation. Genetic analysis from CD138^+^ sorted plasma cells confirmed the presence of the t(6; 14) translocation, further contributing to the complexity of the case.

KD-Venetoclax Treatment: Given the patient’s refractory myeloma, the presence of a plasmacytoma, and the identification of t(6;14) translocation, a decision was made to initiate KD (carfilzomib plus dexamethasone)-Venetoclax (400 mg) therapy in 2021. This therapeutic approach aimed to target the identified genetic abnormality and overcome the patient’s refractoriness to previous treatments. Regular monitoring of treatment response, disease progression, and potential adverse effects was carried out to assess the efficacy and safety of the KD-Venetoclax regimen.

Best Response Ever: Stringent Complete Remission: Remarkably, the patient demonstrated an unprecedented response to the KD-Venetoclax treatment. Serial measurements of the M protein revealed a complete remission, indicating the absence of detectable levels (Figure 1A) and normal free light-chain ratio. This profound response to the KD-Venetoclax regimen represents the best response achieved thus far in the patient’s treatment history. Furthermore, the patient now enjoys ongoing CR for a year without any maintenance therapy, reflecting a very low disease burden achieved with venetoclax-based therapy Such positive outcome offers hope for improved long-term outcomes and underscores the potential effectiveness of targeted therapies tailored to specific genetic abnormalities.

Conclusion: Patient #1, a 56 year-old male with IgG kappa myeloma, faced multiple treatment lines with limited success. The presence of cervical spine plasmacytoma and the identification of t(6;14) translocation posed significant challenges in managing his refractory disease. The initiation of KD-Venetoclax therapy aimed to address these complexities, providing a targeted approach against the identified genetic abnormality. The remarkable sustained complete remission observed following KD-Venetoclax treatment represents a significant milestone in the patient’s journey and highlights the potential of tailored therapies in improving treatment outcomes. Continuous monitoring and further investigation are required to assess the durability and long-term benefits of this exceptional treatment response.

### Case report: patient #2

Patient Information: A 78 year-old male was diagnosed with IgG kappa myeloma in January 2022. The ISS score was determined as I, and FISH analysis from CD138^+^ selected plasma cells revealed an atypical *IGH::CCND3* fusion with the absence of *CCND3::IGH* on the derivative chromosome 6. Notably, three intact *FGFR3* genes (possible trisomy 4) and three intact *CCND1* genes (possible trisomy 11) were also detected by interphase FISH.

Treatment History: The patient initially received VDT (bortezomib-thalidomide-dexamethason) treatment, but not even minimal response (MR) was observed. The patient’s condition and the refractoriness to VDT treatment necessitated salvage treatment. Due to the presence of t(6;14) translocation, the treatment was switched to a combination of VDT with Venetoclax (400 mg) in March 2022 and to a combination of VD (bortezomib and dexamethasone) with Venetoclax (400 mg) from September 2022 to March 2023. Notably, the patient achieved a rapid VGPR concluding into complete immunohematological response following the treatment combination with Venetoclax/dexamethasone (bortezomib was stopped at month 7 of therapy). Serial measurements of the M protein indicating complete remission are shown in Figure 1B.

### Case report: patient #3

Patient Information: A 73 year-old male was diagnosed with IgA lambda myeloma in September 2020, accompanied by significant light chain excretion. The ISS score was determined as II, and FISH analysis revealed the presence of a t(6;14) translocation, as well as gains of chromosomes 1q21, 11, and 17.

Treatment History: The patient initially received VDT. However, due to intolerance to thalidomide (adverse skin reaction), the treatment line was discontinued. From November VD treatment was combined with lenalidomide, however, due to again adverse skin reactions, this treatment line had to be discontinued. From the end of November, VD treatment (bortezomib and dexamethasone) was administered until February. At the end of February 2021, the patient received high-dose cyclophosphamide (HDCy) priming treatment for peripheral stem cell mobilization, and in March 2021 autologous stem cell transplantation (ASCT) was performed with standard melphalan conditioning. The patient achieved a complete immunohematological response following this treatment combination. Serial measurements of the M protein and λFLC indicating a temporal complete remission are shown in Figure 1C. Unfortunately, due to IMID intolerance, the patient was unable to receive lenalidomide maintenance. After a remission period of 10 months, in December 2022 the disease presented with its first relapse (Figure 1C). As a salvage therapy, Venetoclax (400 mg) plus dexamethasone treatment was initiated on January 20, 2022. Partial remission was achieved as indicated by the serial measurements of the M protein and λFLC shown in Figure 1C.

## Discussion

The three cases presented in this report underscore the potential efficacy of venetoclax treatment in myeloma patients with the t(6;14) translocation. These findings further extend the initial observations from the BELLINI study, emphasizing the importance of targeted therapies in specific genetic subtypes of multiple myeloma. Importantly, our study contributes novelty by specifically evaluating the response to venetoclax in patients with the t(6;14) translocation, a relatively understudied area.

The t(6;14) translocation is rarely seen in multiple myeloma, accounting for approximately 1%–3% of cases. This translocation leads to distinct alterations in gene expression profiles. One of the notable consequences of the t(6;14) translocation is the dysregulation of the *CCND3* (Cyclin D3) gene, which is involved in cell cycle regulation. This dysregulation results in increased expression of Cyclin D3, leading to uncontrolled cell proliferation and contributing to the pathogenesis of myeloma with t(6;14) translocation. Prognostically, myeloma patients with t(6;14) translocation tend to exhibit distinct molecular patterns, clinical features, and outcomes compared to those without this translocation. Studies with limited power have suggested that myeloma cases with t(6;14) translocation may be associated with a higher risk of disease progression, shorter survival times, and poorer responses to conventional treatments. The presence of this translocation may correlate with a more aggressive disease course and with a more challenging clinical scenario. Given the unique gene expression profile and uncertain prognosis associated with myeloma harboring t(6;14) translocation, there is a need for targeted therapeutic approaches.

The BELLINI study provided valuable insights into the therapeutic potential of venetoclax in multiple myeloma. It demonstrated promising efficacy and improved progression-free survival in patients receiving venetoclax in combination with standard treatment regimens [2]. However, in multiple myeloma BCL-2 expression is varied significantly across molecular and cytogenetic subgroups in multiple myeloma [26, 27]. As a result, the efficacy of venetoclax is also likely to differ accordingly. Previous studies reported the highest expression of BCL-2 among patients with t(11;14) molecular subtypes (CD1, CD2, ie Cyclin D1 high expressor subgroups 1 and 2). Myeloma patients with translocation t(6;14) also characteristically belong either to the CD1 or CD2 subgroups of the TC (Translocation/Cyclin expression) classification system. There is compelling evidence that venetoclax is unusually effective in patients with the t(11;14) translocation [26–29]. However, neither the BELLINI study nor subsequent similar studies focused specifically on the effects of venetoclax in rare patients with the t(6;14) translocation. The unique aspect of our study lies in its dedicated evaluation of venetoclax treatment in this specific genetic subgroup.

The treatment outcomes observed in our three patients with t(6;14) translocation-associated myeloma provide compelling evidence of the rapid and deep therapeutic responses achieved with venetoclax-based therapies. Patient 1, who had been battling myeloma for nearly a decade and experienced partial responses alternating with relapses during previous treatments, achieved sustained complete remission (CR) when carfilzomib-dexamethasone treatment was combined with venetoclax. Similarly, Patient 2, who exhibited complete refractoriness to frontline VDT therapy, attained CR after transitioning to VD-venetoclax treatment. Patient 3, an IMID-intolerant individual with the t(6;14) translocation, demonstrated a highly favorable response to Venetoclax-Dexamethasone therapy in his first relapse after ASCT. These outcomes highlight the efficacy of venetoclax in this specific genetic subgroup and reinforce its potential as a valuable therapeutic option. Notably, no significant adverse effects were observed in any of the patients.

The collective evidence derived from these cases strongly suggests that venetoclax holds substantial promise as an effective treatment option for myeloma patients harboring the t(6;14) translocation. The notable efficacy of venetoclax in both t(11;14) and t(6;14) translocation subgroups can be attributed to the striking similarity in gene expression profiles observed between these two subgroups. Recent studies suggest that venetoclax-sensitive myeloma cell lines retain a B cell–like pattern of gene expression and chromatin accessibility [26]. It is also likely that gene expression signatures that determine venetoclax sensitivity in myeloma cells are linked to the expression/activity of components of the electron transport chain [28]. The observed treatment responses, such as complete remission and immunohematological response, underscore the potential of venetoclax to overcome refractoriness to prior therapies and deliver significant clinical benefits within these specific genetic subgroups. These patients did not receive CYP3A4 inhibitors to enhance venetoclax blood levels and activity, indicating that in this subgroup of patients the 400 mg daily dose may be sufficient to induce remission. These findings emphasize the critical significance of personalized therapeutic strategies that effectively target specific genetic abnormalities, including the t(6;14) translocation, to optimize treatment outcomes and enhance the overall prognosis for affected patients.

In addition to the treatment outcomes observed in our three patients, our study plays a pivotal role in addressing a significant gap in the existing literature by specifically investigating the efficacy of venetoclax in the context of t(6;14) translocation-associated myeloma. By focusing on this specific genetic abnormality, we provide novel insights into the management of a subgroup that has historically faced limited treatment options. Through our ongoing research, we aim to contribute to the growing body of knowledge surrounding this specific genetic alteration, ultimately advancing the understanding and therapeutic management of t(6;14) translocation-associated myeloma. A comprehensive investigation of venetoclax's efficacy in t(6;14) translocation-associated myeloma will provide valuable information for clinicians and researchers, offering potential new avenues for improving patient outcomes.

It is important to acknowledge certain limitations associated with this retrospective analysis. These include the potential for missing or incomplete data within the medical records and the lack of Bcl-2 expression analysis, which would have provided valuable insight into the relationship between Bcl-2 expression levels and treatment response. Additionally, these case reports represent the experiences of a limited number of patients and may not be entirely generalizable to other individuals with similar conditions. Further studies involving larger patient cohorts and prospective designs are required to confirm and expand upon our findings.

In conclusion, our findings provide preliminary evidence supporting the targeted efficacy of venetoclax in t(6;14) *IGH::CCND3* translocation-associated myeloma, thus extending the existing evidence from the BELLINI study. However, further research and larger studies are necessary to validate these findings and optimize treatment strategies specifically tailored to this genetic subtype. The exploration of personalized approaches targeting specific genetic abnormalities, including the t(6;14) translocation, holds great potential for improving outcomes in patients with multiple myeloma [30–33]. Therefore, when we encounter myeloma patients with an IgH-related translocation lacking an identified partner, pursuing further genetic investigations is of utmost importance. This diligent approach allows us to identify the rare occurrences of t(6;14) translocation, which may constitute only 3% of cases. Nevertheless, such identification presents a valuable opportunity for these individuals to receive highly effective therapies. It is crucial to note that the random application of venetoclax-based therapeutic regimens in other cytogenetic contexts is not recommended in the absence of Bcl-2 expression measurement.

## Data Availability

The raw data supporting the conclusion of this article will be made available by the authors, without undue reservation.

## References

[1] Urban VS Cegledi A Mikala G. Multiple myeloma, a quintessential malignant disease of aging: a geroscience perspective on pathogenesis and treatment. Geroscience (2023) 45:727–46. 10.1007/s11357-022-00698-x 36508077 PMC9742673

[2] Kumar SK Harrison SJ Cavo M de la Rubia J Popat R Gasparetto C Venetoclax or placebo in combination with bortezomib and dexamethasone in patients with relapsed or refractory multiple myeloma (BELLINI): a randomised, double-blind, multicentre, phase 3 trial. Lancet Oncol (2020) 21:1630–42. 10.1016/S1470-2045(20)30525-8 33129376

[3] Bahlis NJ Baz R Harrison SJ Quach H Ho SJ Vangsted AJ Phase I study of venetoclax plus daratumumab and dexamethasone, with or without bortezomib, in patients with relapsed or refractory multiple myeloma with and without t(11;14). J Clin Oncol (2021) 39:3602–12. 10.1200/JCO.21.00443 34388020 PMC8577687

[4] Kaufman JL Gasparetto C Schjesvold FH Moreau P Touzeau C Facon T Targeting BCL-2 with venetoclax and dexamethasone in patients with relapsed/refractory t(11;14) multiple myeloma. Am J Hematol (2021) 96:418–27. 10.1002/ajh.26083 33368455 PMC7986778

[5] Regidor B Goldwater MS Wang J Bujarski S Swift R Eades B Low dose venetoclax in combination with bortezomib, daratumumab, and dexamethasone for the treatment of relapsed/refractory multiple myeloma patients-a single-center retrospective study. Ann Hematol (2021) 100:2061–70. 10.1007/s00277-021-04555-3 33987683

[6] Nguyen N Chaudhry S Totiger TM Diaz R Roberts E Montoya S Combination venetoclax and selinexor effective in relapsed refractory multiple myeloma with translocation t(11;14). NPJ Precis Oncol (2022) 6:73. 10.1038/s41698-022-00315-2 36261486 PMC9581939

[7] Abuelgasim KA Alherz N Alhejazi A Damlaj M. Venetoclax in combination with carfilzomib and dexamethasone in relapsed/refractory multiple myeloma harboring t(11,14)(q13;q32): two case reports and a review of the literature. J Med Case Rep (2020) 14:54. 10.1186/s13256-020-02376-y 32321588 PMC7178736

[8] Basali D Chakraborty R Rybicki L Rosko N Reed J Karam M Real-world data on safety and efficacy of venetoclax-based regimens in relapsed/refractory t(11;14) multiple myeloma. Br J Haematol (2020) 189:1136–40. 10.1111/bjh.16454 32012228 PMC9291136

[9] Boccon-Gibod C Talbot A Le Bras F Frenzel L Royer B Harel S Carfilzomib, venetoclax and dexamethasone for relapsed/refractory multiple myeloma. Br J Haematol (2020) 189:e73–6. 10.1111/bjh.16483 32012229

[10] Costa LJ Davies FE Monohan GP Kovacsovics T Burwick N Jakubowiak A Phase 2 study of venetoclax plus carfilzomib and dexamethasone in patients with relapsed/refractory multiple myeloma. Blood Adv (2021) 5:3748–59. 10.1182/bloodadvances.2020004146 34470049 PMC8679663

[11] Gasparetto C Bowles KM Abdallah AO Morris L Mander G Coppola S A phase II study of venetoclax in combination with pomalidomide and dexamethasone in relapsed/refractory multiple myeloma. Clin Lymphoma Myeloma Leuk (2021) 21:775–84. 10.1016/j.clml.2021.07.029 34551886

[12] He W He F Hu H. Efficacy and safety of Venetoclax-based regimens in relapsed or refractory multiple myeloma: a systematic review and meta-analysis of prospective clinical trials. Ann Med (2023) 55:1029–36. 10.1080/07853890.2023.2186480 36911885 PMC10795640

[13] Kovacs SB Luan J Dold SM Weis A Pantic M Duyster J Venetoclax in combination with carfilzomib, doxorubicin and dexamethasone restores responsiveness in an otherwise treatment-refractory multiple myeloma patient. Haematologica (2020) 105:e138–40. 10.3324/haematol.2019.232330 31467125 PMC7049367

[14] Kumar S Kaufman JL Gasparetto C Mikhael J Vij R Pegourie B Efficacy of venetoclax as targeted therapy for relapsed/refractory t(11;14) multiple myeloma. Blood (2017) 130:2401–9. 10.1182/blood-2017-06-788786 29018077

[15] Sidiqi MH Al Saleh AS Kumar SK Leung N Jevremovic D Muchtar E Venetoclax for the treatment of multiple myeloma: outcomes outside of clinical trials. Am J Hematol (2021) 96:1131–6. 10.1002/ajh.26269 34115387

[16] Swan D Delaney C Natoni A O'Dwyer M Krawczyk J. Successful venetoclax salvage in the setting of refractory, dialysis-dependent multiple myeloma with t(11;14). Haematologica (2020) 105:e141–3. 10.3324/haematol.2019.228338 31753932 PMC7049357

[17] Szita VR Mikala G Kozma A Fabian J Hardi A Alizadeh H Targeted venetoclax therapy in t(11;14) multiple myeloma: real world data from seven hungarian centers. Pathol Oncol Res (2022) 28:1610276. 10.3389/pore.2022.1610276 35295611 PMC8918485

[18] Touzeau C Le Gouill S Mahe B Boudreault JS Gastinne T Blin N Deep and sustained response after venetoclax therapy in a patient with very advanced refractory myeloma with translocation t(11;14). Haematologica (2017) 102:e112–4. 10.3324/haematol.2016.160408 28057737 PMC5394947

[19] Lee R Cho SY Lee DG Choi H Park S Cho BS Infections of venetoclax-based chemotherapy in acute myeloid leukemia: rationale for proper antimicrobial prophylaxis. Cancers (Basel) (2021) 13:6285. 10.3390/cancers13246285 34944903 PMC8699304

[20] Papayannidis C Nanni J Cristiano G Marconi G Sartor C Parisi S Impact of infectious comorbidity and overall time of hospitalization in total outpatient management of acute myeloid leukemia patients following venetoclax and hypomethylating agents. Eur J Haematol (2022) 108:449–59. 10.1111/ejh.13753 35156731 PMC9314138

[21] Zhu LX Chen RR Wang LL Sun JN Zhou D Li L A real-world study of infectious complications of venetoclax combined with decitabine or azacitidine in adult acute myeloid leukemia. Support Care Cancer (2022) 30:7031–8. 10.1007/s00520-022-07126-y 35585204

[22] Bhalla S Melnekoff DT Aleman A Leshchenko V Restrepo P Keats J Patient similarity network of newly diagnosed multiple myeloma identifies patient subgroups with distinct genetic features and clinical implications. Sci Adv (2021) 7:eabg9551. 10.1126/sciadv.abg9551 34788103 PMC8598000

[23] Shaughnessy JD Jr Barlogie B. Using genomics to identify high-risk myeloma after autologous stem cell transplantation. Biol Blood Marrow Transpl (2006) 12:77–80. 10.1016/j.bbmt.2005.10.002 16399589

[24] Sarin V Yu K Ferguson ID Gugliemini O Nix MA Hann B Evaluating the efficacy of multiple myeloma cell lines as models for patient tumors via transcriptomic correlation analysis. Leukemia (2020) 34:2754–65. 10.1038/s41375-020-0785-1 32123307 PMC7483300

[25] Szalat R Avet-Loiseau H Munshi NC. Gene expression profiles in myeloma: ready for the real world? Clin Cancer Res (2016) 22:5434–42. 10.1158/1078-0432.CCR-16-0867 28151711 PMC5546147

[26] Gupta VA Barwick BG Matulis SM Shirasaki R Jaye DL Keats JJ Venetoclax sensitivity in multiple myeloma is associated with B-cell gene expression. Blood (2021) 137:3604–15. 10.1182/blood.2020007899 33649772 PMC8462405

[27] Todoerti K Taiana E Puccio N Favasuli V Lionetti M Silvestris I Transcriptomic analysis in multiple myeloma and primary plasma cell leukemia with t(11;14) reveals different expression patterns with biological implications in venetoclax sensitivity. Cancers (Basel) (2021) 13:4898. 10.3390/cancers13194898 34638381 PMC8508148

[28] Bajpai R Sharma A Achreja A Edgar CL Wei C Siddiqa AA Electron transport chain activity is a predictor and target for venetoclax sensitivity in multiple myeloma. Nat Commun (2020) 11:1228. 10.1038/s41467-020-15051-z 32144272 PMC7060223

[29] Matulis SM Gupta VA Neri P Bahlis NJ Maciag P Leverson JD Functional profiling of venetoclax sensitivity can predict clinical response in multiple myeloma. Leukemia (2019) 33:1291–6. 10.1038/s41375-018-0374-8 30679802 PMC6891824

[30] van Laar R Flinchum R Brown N Ramsey J Riccitelli S Heuck C Translating a gene expression signature for multiple myeloma prognosis into a robust high-throughput assay for clinical use. BMC Med Genomics (2014) 7:25. 10.1186/1755-8794-7-25 24885236 PMC4032347

[31] Zhan F Barlogie B Mulligan G Shaughnessy JD Jr Bryant B. High-risk myeloma: a gene expression based risk-stratification model for newly diagnosed multiple myeloma treated with high-dose therapy is predictive of outcome in relapsed disease treated with single-agent bortezomib or high-dose dexamethasone. Blood (2008) 111:968–9. 10.1182/blood-2007-10-119321 18182586 PMC2200846

[32] Shaughnessy JD Jr Zhan F Burington BE Huang Y Colla S Hanamura I A validated gene expression model of high-risk multiple myeloma is defined by deregulated expression of genes mapping to chromosome 1. Blood (2007) 109:2276–84. 10.1182/blood-2006-07-038430 17105813

[33] Shaughnessy J Jr Zhan F Barlogie B Stewart AK. Gene expression profiling and multiple myeloma. Best Pract Res Clin Haematol (2005) 18:537–52. 10.1016/j.beha.2005.02.003 16026736

